# HDL-associated vitamin D binding protein levels are inversely associated with necrotic plaque burden in psoriasis

**DOI:** 10.1016/j.athplu.2024.12.002

**Published:** 2024-12-13

**Authors:** M.P. Playford, H. Li, A.K. Dey, E.M. Florida, H.L. Teague, S.M. Gordon, N.N. Mehta

**Affiliations:** a Section of Inflammation and Cardiometabolic Diseases, National Heart, Lung and Blood Institute, National Institutes of Health, Bethesda, MD, USA; b Saha Cardiovascular Research Center, University of Kentucky, Lexington, KY, USA; c Department of Physiology, University of Kentucky, Lexington, KY, USA

**Keywords:** Vitamin D binding protein, High density lipoprotein, Psoriasis, Coronary artery disease, Inflammation, Imaging

## Abstract

**Background and aims:**

Vitamin D binding protein (DBP) serves a dual function as a vitamin D carrier and actin scavenger. Free DBP is present in high concentrations in serum, while a smaller pool is bound to lipoproteins like HDL and VLDL. The role of DBP's interaction with lipoproteins remains unclear. Given that HDL has been proposed to have both atheroprotective and anti-inflammatory properties, we sought to compare whether HDL-associated DBP and/or total serum DBP could serve as useful biomarkers for assessing disease severity in psoriasis and cardiovascular disease.

**Methods:**

Psoriasis (PSO) patients (N = 83), which were part of a prospective, observational cohort and non-psoriasis (non-PSO) subjects (n = 35) underwent blood collection for HDL purification by liquid chromatography and CCTA scans to assess coronary plaque burden. Serum and HDL-bound DBP levels were measured by ELISA.

**Results:**

The psoriasis cohort was middle-aged (mean ± IQR: 50 (38–59), predominantly male (n = 55, 66 %) and had moderate-to-severe skin disease [psoriasis area severity index score, PASI score, med (IQR): 9.6 (6–18.3)]. Consistent with our previous reports, PSO patients had significantly higher Framingham Risk Score (FRS), high sensitivity C-reactive protein (hs-CRP), Body Mass Index (BMI), insulin resistance (HOMA-IR) and total coronary plaque burden, driven by the rupture-prone non-calcified necrotic core. However, while the concentration of serum DBP (S-DBP) between PSO and non-PSO was unchanged (PSO: 177.80 (125.77–250.99) vs non-PSO: 177.74 (104.32–254.04), the concentration of DBP associated with HDL (HDL-DBP) was decreased in psoriatics (PSO μg/ml: 1.38 (0.64–2.75) vs non-PSO: 1.72 (1.18–3.90). Although both S-DBP and HDL-DBP levels showed inverse correlations with a measure of skin disease severity (PASI) (S-DBP, Rho = −0.022 vs HDL-DBP, Rho = −113), only HDL-DBP exhibited an inverse relationship with necrotic plaque burden [Rho −0.226, p = 0.085 vs S-DBP (0.041, p = 0.76)]. This relationship was strengthened after adjusting for traditional cardiovascular risk factors such as age and sex (β = −0.237, p = 0.045), FRS (β = −0.295, p = 0.033) and including biological treatment and HDL-cholesterol (β = −0.213, p = 0.048).

**Conclusions:**

In conclusion, we found HDL-DBP levels may better capture the severity of psoriatic disease and association with cardiovascular risk factors than S-DBP.

## Highlights

• HDL-associated Vitamin D Binding protein (HDL-DBP), but not serum DBP (S-DBP) is decreased in patients with psoriasis.
• S-DBP and HDL-DBP have an inverse relationship with LDL particle size.
• HDL-DBP but not S-DBP has an inverse relationship with IL-1β.
• HDL-DBP but not S-DBP has an inverse relationship with necrotic plaque burden even after adjustment with cardiovascular risk factors.

## Graphical abstract

**Figure:**
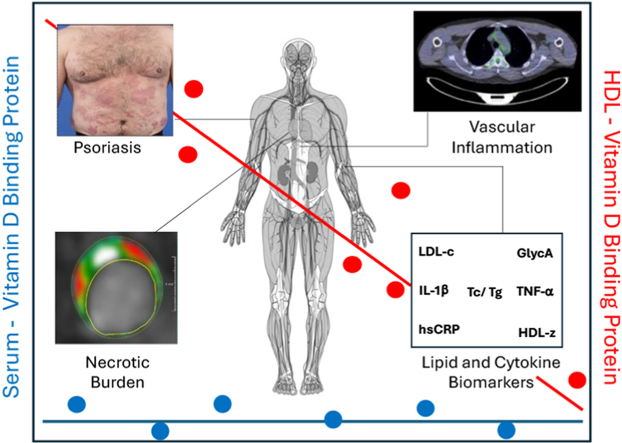

## Abbreviations:

CAD: Coronary artery disease
CCTA: Coronary computed tomography angiography
HDL: High density lipoprotein
NCB: Non-calcified coronary plaque burden
NB: Necrotic Burden
PASI: Psoriasis area severity index
VLDL: Very low density lipoprotein
18F-FDG PET/CT: 18F-labeled fluoro-2-deoxyglucose positron emission tomography/computed tomography

## 1 Introduction

Vitamin D binding protein (DBP) is a highly polymorphic protein that is found in serum at high concentrations (approximately 5 μM) [1]. Numerous studies have established that DBP interacts with actin, fatty acids and both active 1,25 dihyroxyvitamin D (1,25(OH)_2_D) and inactive 25-hydroxyvitamin D (25OHD) metabolites, albeit having a stronger affinity for the latter [1,2]. DBP is the primary transport protein for vitamin D metabolites with approximately 85 % of 25OHD complexed with DBP in the blood [1,2].

High Density Lipoproteins (HDL) interact with a heterogeneous set of proteins that have been proposed to exert numerous antiatherogenic properties, including the passage of cholesterol from the arterial wall to the liver for excretion, termed reverse cholesterol transport (RCT) [3]. A major protein component of HDL is Apolipoprotein A1 (ApoA1) and it is this component which facilitates RCT via interactions with ATP-binding cassette transporter (ABC)-A1, G1 or by the scavenger receptor SR-B1 [3]. There are now nearly 50 studies that have reported over 200 proteins HDL-interacting proteins, and the possible roles of these proteins in HDL functions that are independent of lipid transport have been the subject of intense investigation [3,4]. For example, the HDL proteome includes serine proteases or their inhibitors and paraoxonases [[3], [4], [5], [6]]. While an HDL-associated serine protease inhibitor such as SERPINA3 may regulate inflammation and complement activation, paroxonases, such as PON1 may contribute to the anti-oxidative capacity of HDL [4,5,7]. In both examples mentioned above, there is a proposition that HDL may serve as a carrier for enzymes that otherwise might not reach target sites independently. Finally, DBP has been identified on HDL in over 30 independent studies of the HDL proteome [4]. The role of HDL-associated DBP is unknown, but a delivery of DBP or DBP-bound vitamin D metabolites to target tissues is plausible.

Psoriasis is a chronic inflammatory skin disease that affects an estimated 60 million people worldwide [8,9]. Psoriasis patients, particularly those with severe skin disease, have an increased risk of myocardial infarction, stroke and overall cardiovascular mortality [[10], [11], [12]]. Our laboratory has previously shown that biomarkers in psoriatic disease are associated with high-risk coronary plaque features such as non-calcified burden (NCB) and fibro-fatty burden (FFB) when compared to healthy controls [13,14].

Numerous studies have revealed DBP as a biomarker in both inflammatory and cardiometabolic diseases [[15], [16], [17]]. Serum DBP was found to have lower expression in patients with type 1 diabetes than healthy controls [15]. Similarly, lower serum DBP was also found to be associated with the degree of coronary artery stenosis [16]. In contrast, in a random cohort of 999 participants from the Multi-Ethnic Study of Atherosclerosis (MESA), increasing serum levels of DBP were associated with an increased risk of coronary heart disease events [17]. A compelling case for serum DBP as a biomarker in psoriasis or other skin diseases has not been shown. Additionally, HDL-associated DBP as a biomarker for any human disease has not been reported.

A previous study from our laboratory revealed the specific atheroprotective role of active 1,25(OH)_2_D vitamin D compared to inactive 25OHD [18]. We subsequently questioned whether a protein involved in delivering vitamin D to target tissues such as DBP would have an altered serum profile in psoriasis patients with cardiovascular disease. The further observation that DBP may be HDL-associated prompted a second consideration; whether S-DBP or HDL-DBP would be a more sensitive biomarker in the prediction of psoriatic disease or cardiovascular risk.

## 2 Methods

### 2.1 Study design and Selection of study groups

In a prospective, observational design, 318 participants were recruited in an ongoing cohort study to understand the association between psoriasis and cardiometabolic disease under

The Psoriasis, Atherosclerosis and Cardiometabolic Initiative (PACI; NCT01778569) from January 2013 through April 30, 2019. Of the 318 potential participants, 83 consecutive psoriasis patients were included in our analyses who had a combination of available CCTA, sufficient blood volume, and a one-year follow-up visits. In addition, a cohort of healthy volunteers in an ongoing longitudinal prospective study (ICKD; NCT01934660) from December 2013 to March 2020 was included as controls. Strengthening the reporting of observational studies in epidemiology guidelines were followed for reporting the findings. All patients provided informed consent before study participation. Detailed inclusion and exclusion criteria of psoriasis participants are previously described [18].

### 2.2 Clinical assessment

All visiting patients were seen at the NIH Clinical Center, (Bethesda, MD, USA) where they received a detailed history and physical exam. All patients underwent 18-FDG PET/CT (18F- fluorodeoxyglucose Positron Emission Tomography) imaging and laboratory testing. Coronary Computed Tomography Angiography (CCTA) scans were acquired on all patients who provided consent and lacked contraindications. A study provider confirmed the onset and duration of psoriasis and assessed psoriasis severity using the Psoriasis Area and Severity Index (PASI) score, which combines the severity of lesions and the area affected into a single score, considering erythema, induration, and desquamation within each lesion.

### 2.3 Coronary artery imaging

All subjects underwent CCTA on the same day as the blood draw, utilizing the same CT scanner (320-dectector row Aquilion ONE ViSION, Toshiba, Japan). Radiation exposure was in accordance with the National Institutes of Health Radiation Exposure Committee guidelines. Images were acquired at a slice thickness of 0.5 mm with a slice increment of 0.25 mm. Coronary artery burden adjusted for luminal attenuation was determined across each of the three main coronary arteries using a semi-automated software QAngio CT (Medis, Netherlands) [19]. Manual adjustment of inner lumen and outer vessel wall delineations was performed if needed. Total coronary artery burden (TB) and non-calcified coronary artery burden (NCB) indices (mm^2^) were calculated by dividing total vessel plaque volume by total vessel length. Total plaque burden was defined as the sum of calcified plaque burden and non-calcified plaque burden. Non-calcified plaque subcomponents, including fibro-fatty (FFB) and necrotic (NB) burdens, were obtained after adaptively correcting for lumen attenuation and depicted using Hounsfield Units.

### 2.4 Measurement of cytokines, vitamin D binding protein, Apolipoprotein A1 and other biomarkers

All measurements were performed in blood collected and centrifuged within 2 h of collection at 2400RPM for 30 min at room temperature (20–23 °C). The serum supernatant was stored at −80 °C until further use.

Plasma levels of IL17a, TNFα, IL1β, MCP-1 and IFNγ were measured using customizable human U-plex kits (Mesoscale Discovery, Gaithersburg, MD, USA) following manufacturer's instructions. Clinical Measurements (GlycA, lipoprotein subclass categories) were measured using the automated Vantera clinical NMR analyzer (Labcorp, NC, USA). High-sensitivity CRP (hsCRP) was measured in the NIH clinical center. Measurements of Cholesterol Efflux were as previously described [20]. Western blotting techniques to detect Apolipoprotein A1 was performed using a rabbit monoclonal antibody (clone EP1368Y) #ab52945 (Abcam, Cambridge, MA, USA). Quantification of Vitamin D Binding Protein in serum and HDL was performed by ELISA #DVDBP0B (R and D Systems, Minneapolis, MN, USA) following manufacturer's instructions with each serum sample being diluted 1:2000 in diluent RD5P. HDL was assayed undiluted. This assay kit detects the three most abundant polymorphisms of DBP (Gc1f, Gc1s and Gc2) with equal affinity [[21], [22], [23]]. A white paper demonstrating equal allele detection can be provided at (https://www.rndsystems.com/product-highlight/improved-sensitivity-vitamin-d-bp-elisa-kits).

### 2.5 Purification of high density lipoprotein

Serum was thawed and 0.4 ml was injected on an NGC FPLC system (Biorad, Hercules, CA, USA) and separated over two Enrich™ SEC 650 10x300 columns cat#7801650 (Biorad) arranged in series at a flow rate of 0.5 ml/min in Tris buffer (10 mM Tris, 0.15M NaCl, 1 mM EDTA). Typically, 60 fractions of 0.5 ml were collected and stored at 4 °C. Vitamin D binding protein content in the HDL fractions was measured by ELISA after less than 30 days storage. FPLC fractions were tested for phospholipid content using enzymatic kits from Wako Diagnostics (Richmond, VA). The protein concentration of HDL fractions was measured by.

Bicinchoninic acid (BCA) kit (cat#23225; ThermoFisher Scientific, Waltham, MA, USA). Removal of lipoprotein fractions was performed using Cleanascite™ lipid removal reagent cat #X2555-10 (Biotech Support Group, Monmouth Junction, NJ, USA) following the manufacturer's recommended instructions.

### 2.6 Statistical analysis

Data were reported as mean with standard deviation for parametric variables, median with interquartile range (IQR) for non-parametric variables and percentages for categorical variables. In baseline analyses, parametric and non-parametric variables were compared between the two groups using student t-test and Mann-Whitney *U* test, respectively. The association between S-DBP and HDL-DBP with cardiometabolic and inflammatory parameters was assessed by Spearman correlation. The relationship of S- and HDL-DBP with cardiovascular burden was analyzed using univariable and multivariable regression with potential confounders. Standardized beta values from these analyses were reported, which indicate number of standard deviations change in the outcome variable per standard deviation change in the predicting variable. P-value<0.05 was deemed significant. All statistical analyses were performed using R version 4.3.2.

## 3 Results

### 3.1 Isolation of HDL from human serum and identification of HDL-containing DBP

**Fig. 1:**
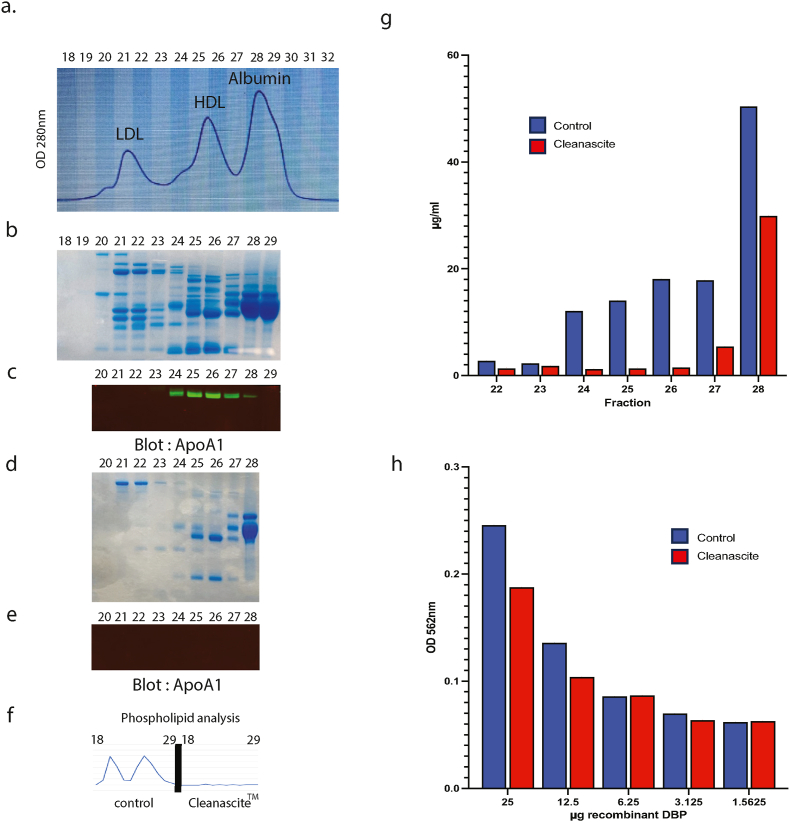
**Identification of DBP in isolated High Density Lipoproteins** Elution profiles from two NGC size exclusion columns in series (a). Collected fractions (18–29) were separated by SDS-PAGE gel (4–12 % NuPAGE™ Bis-Tris, Thermo Fisher Scientific) and stained with Coomassie blue (SimplyBlue™ Safestain, Thermo Fisher Scientific) (b). A second SDS-PAGE gel using the fractions in (b) was probed with a monoclonal antibody to Apolipoprotein A1 (c). FPLC fractions (20–28) in (b) were treated with Cleanascite™ lipid removal agent, separated with by SDS-PAGE gel (4–12 % NuPAGE™ Bis-Tris, Thermo Fisher Scientific) and stained with Coomassie blue (d). A second SDS-PAGE gel using the Cleanascite™ treated fractions in (d) was probed with a monoclonal antibody to Apolipoprotein A1 (e). Phospholipid analysis of FPLC fractions (18–29) ± Cleanascite™ treatment (f). FPLC fractions (22–28) ± Cleanascite™ treatment assessed for DBP levels by ELISA (g). The indicated amounts of recombinant DBP were treated with Cleanascite™ or phosphate-buffered saline control. Supernatent was isolated and assessed for DBP levels by ELISA (h).

As a first step into testing the role of DBP as a biomarker in psoriasis and related comorbidities, HDL was isolated from patient serum. To this end, a widely known gel-fitration method was used to fractionate proteins into three distinct peaks, representing LDL, HDL, and albumin (Fig. 1a). Protein content was found in fractions 19–29 with the most highly concentrated protein of approximately 50kD found in fractions 28 and 29, encompassing the third peak which was expected to be albumin (Fig. 1b). The middle peak was confirmed to be HDL, by the presence of Apolipoprotein A1 which encompassed fractions 24 to 28 but was most highly abundant in fractions 25 and 26 (Fig. 1c).

Next, we sought to determine whether DBP was found in HDL particles or just as a co-fractioning protein from chromatography. Fractions 20 to 28 were treated with a lipid-removal agent (Cleanascite™), and while this treatment removed many of the proteins in fractions 21 to 27, the non-lipidated ‘albumin’ fraction remained (Fig. 1d). In contrast, ApoA1 was not detected in protein fractions treated with the lipid-removal reagent (Fig. 1e). Furthermore, phospholipid analysis of fractions 18 to 29 revealed that 21, 22 and 25, 26 are rich in phospholipid and were removed with Cleanascite™ treatment (Fig. 1f). Finally, the presence of DBP in fractions 22 to 28 was assessed by Enzyme-linked immunosorbent assay (ELISA). DBP was readily detected in fractions 24 to 27, but mostly removed upon lipid-removal. In the same conditions, DBP was abundantly detected in fraction 28 suggesting that in this fraction, DBP is not bound to lipidated proteins (Fig. 1g). The fact that DBP has similar protein molecular weight as albumin (50kD) likely explains the detection of DBP in this fraction.

To verify that the removed DBP was HDL-bound, this lipid-removal agent was also incubated with recombinant DBP (at a range of 1–25 μg) and ELISA was performed on the remaining supernatant (Fig. 1h). At all tested concentrations of DBP, most remained unbound to the lipid removal agent demonstrating that DBP does not bind the Cleanascite™ in the absence of lipid. Taken together, this set of analyses revealed that the most concentrated fractions of HDL were determined to be 25 and 26, and that these two fractions contain DBP. These two fractions were subsequently pooled to assess for DBP content.

### 3.2 Comparison of serum- and HDL-DBP levels in psoriasis patients and control subjects

Vitamin D Binding Protein (DBP) is an abundant protein in human serum and also contained within an HDL molecule. Using a well-characterized cohort of psoriasis patients, we questioned which DBP pool is superior as a biomarker for psoriasis and associated comorbidities. The psoriasis cohort (N = 83) was middle-aged [mean ± IQR: 50 (38–59)], predominantly male (n = 55, 66 %) and had moderate-to-severe skin disease [psoriasis area severity index score, PASI score, med. (IQR): 9.6 (6–18.3)], with less than a quarter on biologic psoriasis therapy for skin disease management (n = 13 (anti-TNFα n = 10, anti-IL17 n = 2, anti-IL12/23 n = 1)) at baseline (Table 1). Psoriasis patients had a low cardiovascular risk by Framingham 10-year risk (FRS) [1.8 (0.4–4.93)], despite a third having a history of dyslipidemia (n = 27, 33 %) (Table 1). A control cohort (n = 35) of non-psoriasis subjects were younger than the psoriasis cohort [mean ± IQR: 34 (24–48)], had a similar proportion of male subjects (n = 19, 61 %) and as expected also had a lower FRS [0.35 (0.1–1.35)] (Table 1).

**Table 1:** Baseline characteristics for psoriasis patients versus control subjects.

Parameter	Non-Psoriasis	Psoriasis	P-value
	(N = 35)	(N = 83)	
**Demographic and Clinical Characteristics**			
Age, years	34 (24–48)	50 (38–59)	**<0.001**
Males n (%)	19 (61)	55 (66)	0.78
Hypertension. n (%)	1 (3)	20 (24)	**0.013**
Hyperlipidemia n (%)	5 (14)	27 (33)	0.07
Type-2 diabetes n (%)	1 (3)	7 (8)	0.43
Current smoker n (%)	0 (0)	8 (10)	0.11
FRS	0.35 (0.1–1.35)	1.8 (0.4–4.93)	**0.007**
**Clinical and Lab Values**			
Total cholesterol, mg/dL	173 (146–204)	180 (152–209)	0.46
HDL cholesterol, mg/dL	58 (50–68)	50 (42–60)	**0.004**
LDL cholesterol, mg/dL	98 (74–115)	107 (82–134)	0.047
Triglycerides, mg/dL	80 (65–109)	92 (66–136)	0.26
hs-CRP, mg/L	0.6 (0.4–1.1)	1.8 (0.8–5.0)	**<0.001**
Body mass index	24.1 (22.5–25.9)	29.2 (24.9–34.4)	**<0.001**
Waist to hip ratio	0.92 (0.84–0.98)	0.99 (0.91–1.03)	**<0.001**
Glucose mg/dL	92 (85–96)	95 (89–105)	**0.02**
HOMA-IR	1.54 (0.89–2.35)	2.79 (1.45–5.13)	**<0.001**
Serum DBP (μg/ml)	177.74 (104.32–254.04)	177.80 (125.77–250.99)	>0.99
HDL- DBP (μg DBP/mg HDL)	1.72 (1.18–3.90)	1.38 (0.64–2.75)	0.095
**Psoriasis Characterization**			
PASI Score		9.6 (6–18.3)	
Biologic treatment		13 (16)	
**CCTA Characterization**			
Total burden, mm2 (x100)	0.91 (0.80–1.06)	1.14 (0.86–1.52)	**0.008**
Non-calcified burden, mm2 (x100)	0.90 (0.75–1.06)	1.11 (0.81–1.43)	**0.020**
Dense calcified burden, mm2 (x100)	0.01 (0.01–0.02)	0.03 (0.01–0.07)	**0.003**

Furthermore, consistent with our previous work, the psoriasis cohort had elevated inflammation [hsCRP, mg/L, (IQR): psoriasis 1.8 (0.8–5) vs non-psoriasis 0.6 (0.4–1.1), p < 0.001] and coronary plaque burden [total burden, mm^2^ x100, (IQR): psoriasis 1.14 (0.86–1.52) vs non-psoriasis 0.91 (0.8–1.06), p < 0.008] than the control cohort [14]. However, the levels of serum-DBP (sDBP) were similar between psoriasis patients and control subjects [sDBP, μg/ml, (IQR): psoriasis 177.80 (125.77–250.99) vs non-psoriasis 177.74 (104.32–254.04), p > 0.99 (Table 1). In contrast, the levels of HDL-DBP were found to be lower in psoriasis patients than control, [HDL-DBP, μg DBP/mg HDL, (IQR): psoriasis 1.38 (0.64–2.75) vs non-psoriasis 1.72 (1.18–3.90), p = 0.095 (Table 1).

We were concerned that the lower levels of HDL-DBP observed in psoriasis patients may be due to the elevated age of this cohort. Hence, we generated a subset of the entire cohort where psoriasis and non-psoriasis patients were suitably age and sex matched (Supp. Table 1). Both cohorts were approaching middle aged [mean ± IQR: psoriasis 40 (32–54) vs non-psoriasis 38 (25–50)], with a similar proportion of male (psoriasis n = 30, 65 % vs non-psoriasis 18, 57 %). Psoriasis severity remained moderate-to-severe [psoriasis area severity index score, PASI score, med. (IQR): 8.9 (6.3–16.3)], with 17 % (8 of 46) on biologic therapy for skin disease management at baseline (Supp. Table 1). As expected, these younger psoriasis patients had lower FRS [0.6 (0.14–1.99)], which was not significantly different to control subjects [0.4 (0.1–1.3); p = 0.31], but had significantly higher BMI (psoriasis [29.4 (26.0–34.5)] vs non-psoriasis [24.5 (23.0–26)]); p < 0.001. This subset of the psoriasis cohort had elevated pro-inflammatory hs-CRP, (psoriasis [1.8 (0.8–4.8)] vs non-psoriasis [0.7 (0.5–1.3)] mg/dL; p < 0.001, and notably, a slight decrease in HDL-cholesterol (psoriasis [50 (41–60)] vs non-psoriasis [58 (49–70)]mg/dL) which was statistically significant (psoriasis [50 (41–60)] vs non-psoriasis [58 (49–70)]); p = 0.001 (Supp. Table 1). Finally, consistent with the entire cohort, coronary plaque burden was elevated in psoriasis [total burden, mm^2^ x100, (IQR): psoriasis 1.17 (0.96–1.58) vs non-psoriasis 0.97 (0.8–1.08), p = 0.005] than the control cohort. The levels of serum-DBP (s-DBP) were also similar between psoriasis patients and control subjects [sDBP, μg/ml, (IQR): psoriasis 181.43 (127.77–250.99) vs non-psoriasis 183.90 (104.32–254.04), p = 0.77 (Table 1). However, in this age- and sex-matched cohort, the differences in HDL-DBP levels between psoriasis patients and control subjects, were consolidated and significant [HDL-DBP, μg DBP/mg HDL, (IQR): psoriasis 1.12 (0.61–2.16) vs non-psoriasis 1.98 (1.29–5.02), p = 0.014 (Supp.Table 1).

### 3.3 Association of S-DBP and HDL-DBP levels with cardiometabolic and inflammatory parameters

Given that DBP is a transporter of vitamin D, a known anti-inflammatory metabolite, we questioned whether the expression of either S-DBP or HDL-DBP had an association with cardiometabolic and inflammatory parameters (Table 2). Using our deeply phenotyped psoriasis cohort, we used Spearman's correlation coefficient to compare associations of S-DBP and HDL-DBP levels with basic clinical and lab values, inflammatory cytokines, HDL function, vascular inflammation and coronary artery characteristics (Table 2). As expected, both S-DBP and HDL-DBP were inversely correlated with PASI score (S-DBP = −0.022, HDL-DBP = −0.113) but did not reach statistical significance (Table 2). An association with Age, Hypertension, Hyperlipidemia, SBP, DBP, FRS, T2D, Triglycerides and HDL cholesterol, HDL particle size or number were not observed, yet unexpectedly, an inverse correlation between both S-DBP and HDL-DBP and LDL particle size was noted and statistically significant (S-DBP:rho = −0.227, p = 0.039; HDL-DBP:rho = −0.244, p = 0.026). When comparing the relationship between DBP and inflammatory cytokines, we found an inverse relationship between both S-DBP and HDL-DBP and IL-1β, which approached significance with HDL-DBP (S-DBP:rho = −0.095, p = 0.459; HDL-DBP:rho = −0.245,p = 0.053). A relationship between DBP levels and all other tested cytokines was not observed (Table 2).

**Table 2:** Comparisons of the association between S-DBP or HDL-DBP with cardiometabolic and inflammatory parameters of psoriasis patients.

Parameter	S-DBP			HDL-DBP		
Demographic and Clinical Characteristics	N	∗Rho	p-value	N	∗Rho	p-value
HDL-DBP	83	−0.004	0.973			
Age, y	83	0.113	0.311	83	0.032	0.774
Sex, male	83	−0.066	0.554	83	−0.02	0.856
PASI score	81	−0.022	0.843	81	−0.113	0.316
Biologic	83	0.176	0.112	83	−0.196	0.075
BMI	82	0.197	0.076	82	−0.079	0.481
Waist-to-Hip Ratio	79	0.178	0.118	79	0.148	0.194
Hypertension, No.	83	0.072	0.516	83	−0.071	0.526
Hyperlipidemia, No.	83	−0.077	0.487	83	0.158	0.154
Type 2 Diabetes (T2D), No.	83	0.083	0.454	83	0.116	0.297
Framingham Risk Score	81	0.122	0.276	81	−0.019	0.87
Metabolic Syndrome, No.	83	0.206	0.062	83	0.073	0.51
Systolic blood pressure (SBP), mm Hg	82	0.145	0.195	82	0.074	0.51
Diastolic blood pressure (DBP), mm Hg	82	0.228	0.039	82	−0.008	0.941
**Clinical and Lab Values**						
Total cholesterol, mg/dL	83	−0.018	0.869	83	−0.05	0.657
HDL cholesterol, mg/dL	83	0.069	0.537	83	0.074	0.507
HDL particle number	83	0.148	0.182	83	0.201	0.069
HDL particle size, nm	82	0.106	0.343	82	−0.069	0.536
LDL cholesterol, mg/dL	83	−0.038	0.732	83	−0.066	0.551
LDL particle number	83	0.013	0.905	83	0.028	0.803
LDL particle size, nm	83	−0.227	**0.039**	83	−0.244	**0.026**
Triglycerides, mg/dL	83	0.1	0.367	83	0.157	0.155
HOMA-IR	82	0.192	0.083	82	0.089	0.424
Glyca	82	0.295	0.007	82	0.011	0.925
hsCRP, mg/dL	83	0.164	0.139	83	−0.154	0.164
HDL cholesterol efflux capacity	83	−0.031	0.784	83	0.184	0.095
IL-1β	63	−0.095	0.459	63	−0.245	**0.053**
IL-17a	66	−0.229	0.064	66	−0.148	0.236
MCP1	66	−0.121	0.334	66	−0.214	0.084
IFN-γ	66	0.063	0.614	66	−0.171	0.17
TNF-α	66	−0.200	0.108	66	−0.052	0.679
**PET/CT Parameter**						
Bone marrow SUV max	64	0.047	0.713	64	−0.142	0.264
Spleen SUV max	64	0.004	0.972	64	−0.146	0.251
Liver SUV max	64	0.004	0.976	64	−0.082	0.52
**Coronary Artery Characteristics**						
Total coronary burden (TCB), mm^2^ (x100)	78	0.2	0.08	78	−0.076	0.509
Noncalcified coronary burden (NCB), mm^2^ (x100)	78	0.21	0.065	78	−0.068	0.553
Dense calcified burden (DCB), mm^2^ (x100)	78	−0.04	0.73	78	0.015	0.897
Fibrofatty burden (FFB)	75	0.104	0.376	75	−0.104	0.374
Necrotic burden (NB)	59	−0.041	0.76	59	−0.226	**0.085**

∗All data in the table is expressed as Spearman's rank correlation coefficient.

We previously reported an increase in splenic and bone marrow uptake of ^18^F-FDG in psoriasis subjects [24]. In addition, an inverse relationship between the active form of vitamin D (1,25(OH)2D) and aortic vascular uptake of ^18^F-FDG was found [18]. As a transporter for vitamin D, we hypothesized that the levels of DBP would be related to ^18^F-FDG uptake in the indicated organs. However, an association between S-DBP and bone marrow, spleen or liver ^18^F-FDG uptake was not found, though an inverse trend of HDL-DBP levels with all of bone marrow (rho = −0.142), spleen (rho = −0.146) and liver (rho = −0.082) ^18^F-FDG uptake was observed, but not statistically significant (Table 2).

Patients with psoriasis also have elevated coronary plaque burden as determined by CCTA [14]. This imaging method can also identify vulnerable non-calcified plaque components such as fibro-fatty and necrotic burden, the latter of which was shown to be reduced in psoriatics undergoing biologic treatment over one year [25]. Hence, we used these measures of vulnerable plaque to assess the relationship with DBP expression in our psoriasis cohort (Table 2). While an association between S-DBP and all tested coronary artery plaque characteristics was not found, an inverse association between HDL-DBP and all vulnerable plaque characteristics was observed (TB: rho = −0.076, DCB: rho = −0.068, FFB: rho = −0.104, NB: rho = −0.085), but only the relationship between NB and HDL-DBP approached statistical significance (p = 0.085). This relationship was consolidated and reached statistical significance when adjusted for cardiovascular risk factors such as Framingham Risk Score (FRS) alone (natural log unadjusted β = −0.266, p = 0.052 vs FRS β = −0.295, p = 0.033) or in combination with biological treatment (β = −0.326, p = 0.020) (Table 3b). In contrast, the relationship between Necrotic Burden and HDL-DBP diminished slightly with adjustment for age (β = −0.224, p = 0.090) and BMI (Table 3b). Significant associations between S-DBP and NB were not observed when adjusted to cardiovascular risk factors (Table 3a). In conclusion, we show that HDL-DBP is a more sensitive biomarker than S-DBP in predicting measures of vulnerable coronary plaque such as necrotic burden.

**Table 3a:** Regression analysis of Serum-DBP with cardiovascular risk factors.

Model	Std β	P-value
Necrotic Burden		
Unadjusted Adjusted for Age Adjusted for Age, Sex Adjusted for Age, Sex and Body Mass Index (BMI) Adjusted for Age, Sex, BMI and Biological Treatment Adjusted for Age, Sex, BMI, Biological Treatment and HDL-c Adjusted for Age, Sex, BMI, Biological Treatment and HDL particle number Adjusted for Framingham Risk Score (FRS) Adjusted for FRS and Biological Treatment	0.041 0.017 0.074 −0.009 −0.014 0.001 0.033 0.028 0.036	0.770 0.895 0.542 0.944 0.908 0.991 0.772 0.843 0.798

∗Necrotic Burden and Serum DBP are log-transformed.

**Table 3b:** Regression analysis of HDL-DBP with cardiovascular risk factors.

Model	Std β	P-value
Necrotic Burden		
Unadjusted Adjusted for Age Adjusted for Age, Sex Adjusted for Age, Sex and Body Mass Index (BMI) Adjusted for Age, Sex, BMI and Biological Treatment Adjusted for Age, Sex, BMI, Biological Treatment and HDL-c Adjusted for Age, Sex, BMI, Biological Treatment and HDL particle number Adjusted for Framingham Risk Score (FRS) Adjusted for FRS and Biological Treatment Adjusted for FRS and Biological Treatment. HDL-c	−0.266 −0.224 **−0.237** −0.212 **−0.240** **−0.213** **−0.270** **−0.295** **−0.326** **−0.263**	0.052 0.090 **0.045** 0.062 **0.031** **0.048** **0.038** **0.033** **0.020** **0.035**

∗Necrotic Burden and Serum DBP are log-transformed.

## 4 Discussion

Vitamin D binding protein (DBP) is found at micromolar concentrations in the circulation and contained within the HDL molecule. Using a longitudinal study design in a deeply phenotyped cohort of psoriasis patients, the sensitivity of serum or HDL-containing DBP as a biomarker for skin disease severity with cardiometabolic comorbidities and inflammatory cytokines was compared. We observed the following: i) serum DBP (S-DBP) is similar in concentration between control subjects and psoriasis patients, ii) HDL-containing DBP (HDL-DBP) is lower is psoriasis patients than control subjects and iii) both S-DBP and HDL-DBP levels are inversely associated with the size of a LDL particle. Furthermore, HDL-DBP was found to have stronger inverse relationship with circulating IL1β and necrotic burden than S-DBP, which reached statistical significance when adjusted for Framingham Risk Score and those patients receiving biologic therapy.

The role of DBP in inflammatory diseases is not fully understood, with an accurate assessment perhaps being clouded by a combination of numerous genetic polymorphisms and varying detection methods [21,22]. Two previous studies from the same research group, with under 50 psoriasis patients have indicated a small increase of serum DBP compared to healthy controls [26,27], a finding we expected to observe using similar detection methodology. However, we found no difference in S-DBP between psoriasis (median PASI score = 9.6) and non-psoriasis subjects.

In contrast, using a well-characterized chromatographic method to separate HDL from serum, we firstly confirmed the presence of DBP in the HDL molecule [4,28], and surprisingly noted a decrease in DBP contained within the HDL molecule in our psoriasis cohort. While, the expression of HDL-DBP in psoriasis was inversely related to PASI score, numerous inflammatory cytokines and inflammation in the bone marrow, spleen and liver the study was likely inadequately powered for the relationship to be statistically significant (e.g. PASI score rho = 0.113, p = 0.316). We previously reported that serum levels of active 1,25(OH)2D are inversely related to PASI score [18]. Given that DBP is a major transporter of all vitamin D forms, and that both DBP and 25(OH)D have been detected in the Very Low Density Lipoprotein (VLDL) molecules [22,29], we hypothesized that consequently there would be a deficiency of vitamin D carried by DBP in the HDL molecule in psoriasis. However, using the methodology described in Ref. [18] we were unable to detect any vitamin D forms. Given that less than 5 % of total DBP protein has vitamin D bound at any given time, if a similar proportion is carried by HDL-DBP is likely below the threshold of detection [30].

Unexpectedly, in our psoriasis cohort, both S- and HDL-DBP were found to be inversely associated with LDL particle size. In the same cohort, we have previously reported a robust association of small LDL (s-LDL) with non-calcified plaque burden, a marker of subclinal atherosclerosis [31]. LDL size may be determined by multiple metabolic pathways including lipolysis of intermediate-density lipoproteins (IDL), remodeling of LDL by cholesterol ester transfer protein (CETP) or triglyceride hydrolysis in the LDL molecule [32]. While further investigations are warranted to understand the relationship between S-DBP and LDL size, previous reports of a positive relationship between S-DBP and triglyceride concentration offers a little weight to the hypothesis that DBP is involved in LDL metabolism via triglyceride hydrolysis [29].

It has been previously established that the psoriasis cohort used in this study had elevated coronary plaque burden [14], and more recently, that small dense LDL-c (sdLDL-c) may predict vulnerable coronary plaques [13]. In contrast, here we show that HDL-DBP is associated with protection against a feature of vulnerable coronary plaque, necrotic burden. Formation of necrotic plaque regions may occur via several regulated pathways such as pyroptosis, with an end consequence being the activation of IL1β [33]. As HDL-DBP is negatively associated with both IL1β and necrotic burden prompts speculation that the inhibition of pyroptosis may be the mechanism by which HDL-DBP is atheroprotective. Notably, a previous report [34] has examined associations between the HDL proteome and stable (calcified) or vulnerable (non-calcified) plaque in over 100 patients with a clinical indication for coronary CT angiography. Rather than a negative association with vulnerable plaque as demonstrated in this study, the authors found a positive association of HDL-DBP with stable plaque [34]. Taken together, both studies provide evidence of a beneficial impact of HDL-DBP on coronary plaque characteristics.

An unanswered question from this study on our psoriatic patients is whether biologic or systemic therapeutic intervention affects HDL-DBP expression and the relationship with coronary plaque burden. Although the number of suitable patients precluded investigation in this study, of note is work by Holzer et al. which observed both an increased phospholipid content of HDL as well as HDL-bound paraoxonase (PON1) activity in patients treated with anti-psoriatic therapy [35]. HDL-DBP levels were not addressed and hence changes upon therapeutic intervention should not be ruled out.

Changes in the HDL proteome have been observed upon therapeutic intervention in two other studies [36,37]. The effect of niacin therapy on the HDL proteome, found significant changes in HDl-bound Apolipoproteins L1 and A2, Clusterin, Serum Amyloid A and Angiotensinogen [36]. Similarly, the effect of metformin on the proteome of youth type 1 diabetic patients revealed significant changes in peptidoglycan recognition protein 2 and alpha-2-macroglobulin [37].

In summary, the data suggests that HDL-DBP may be both protective for inflammatory diseases and vulnerable coronary plaque. To our knowledge, this is the first study to simultaneously compare the expression of an individual protein in both serum and HDL in an inflammatory disease state. Due to DBP's potential to bind lipids, actin and vitamin D, each of which have a role in CVD, makes it a compelling biomarker warranting further investigation.

## 5 Disclosures

Dr. Mehta is a full-time US government employee and has served as a consultant for Amgen, Eli Lilly, and Leo Pharma receiving grants/other payments; as a principal investigator and/or investigator for AbbVie, Celgene, Janssen Pharmaceuticals, Inc, and Novartis receiving grants and/or research funding; and as a principal investigator for the National Institute of Health receiving grants and/or research funding.

All other authors declare no conflicts of interests in relation to the work presented in this manuscript.

## Funding

This study was supported by the National Heart, Lung and Blood Institute (NHLBI) Intramural Research Program (HL006193- 05).

**Role of theFunder**: The funding source had no role in the design and conduct of the study; collection, management, analysis, and interpretation of the data; preparation, review, or approval of the manuscript; and decision to submit the manuscript for publication.

## Declaration of competing interest

The authors declare the following financial interests/personal relationships which may be considered as potential competing interests: Dr. Mehta is a full-time US government employee and has served as a consultant for Amgen, Eli Lilly, and Leo Pharma receiving grants/other payments; as a principal investigator and/or investigator for AbbVie, Celgene, Janssen Pharmaceuticals, Inc, and Novartis receiving grants and/or research funding; and as a principal investigator for the National Institute of Health receiving grants and/or research funding.
