# Supplementary material for: HDL-associated vitamin D binding protein levels are inversely associated with necrotic plaque burden in psoriasis
Source: Atheroscler Plus. 2024 Dec 13;59:32–8. doi: 10.1016/j.athplu.2024.12.002 (PMC11732513; doi:10.1016/j.athplu.2024.12.002)
Supplement: Multimedia component 1 [file mmc1.docx]

**Supplemental Table 1.**

Baseline characteristics of a subset of psoriasis patients versus control subjects **matched by age**.

| **Parameter** |  |  | | |
| --- | --- | --- | --- | --- |
|  |  | Non-Psoriasis | Psoriasis | P-value |
|  |  | (N=27) | (N=46) |  |
| **Demographic and Clinical Characteristics** |  |  |  |  |
| Age, years |  | 38 (25 - 50) | 40 (32 - 54) | 0.21 |
| Males |  | 18 (67) | 30 (65) | >0.99 |
| Hypertension |  | 1 (4) | 10 (22) | **0.046** |
| Hyperlipidemia |  | 3 (11) | 11 (24) | 0.30 |
| Type-2 diabetes |  | 1 (4) | 2 (4) | >0.99 |
| Current smoker |  | 0 (0) | 3 (7) | 0.55 |
| FRS |  | 0.4 (0.1 - 1.3) | 0.6 (0.14 - 1.99) | 0.31 |
| **Clinical and Lab Values** |  |  |  |  |
| Total cholesterol, mg/dL |  | 181 (147 - 204) | 171 (147 - 211) | 0.88 |
| HDL cholesterol, mg/dL |  | 58 (49 - 70) | 50 (41 - 60) | **0.001** |
| LDL cholesterol, mg/dL |  | 98 (71 - 117) | 113 (87 - 136) | 0.09 |
| Triglycerides, mg/dL |  | 80 (67 - 104) | 88 (66 - 141) | 0.47 |
| hs-CRP, mg/L |  | 0.7 (0.5 - 1.3) | 1.8 (0.8 - 4.8) | **<0.001** |
| Body mass index |  | 24.5 (23.0 - 26.0) | 29.4 (26.0 - 34.5) | **<0.001** |
| Waist to hip ratio |  | 0.93 (0.87 - 0.99) | 0.99 (0.91 - 1.03) | **<0.001** |
| Glucose mg/dL |  | 92 (85 - 95) | 91 (87 - 98) | 0.47 |
| HOMA-IR |  | 1.43 (0.77 - 2.35) | 2.72 (1.45 - 5.08) | **<0.001** |
| Serum DBP (µg/ml) |  | 183.90 (104.32 - 254.04) | 181.43 (127.57 - 250.99) | 0.77 |
| HDL-DBP (µg DBP/mg HDL) |  | 1.98 (1.29 - 5.02) | 1.12 (0.61 - 2.16) | **0.014** |
| **Psoriasis Characterization** |  |  |  |  |
| PASI Score |  |  | 8.9 (6.3 – 16.3) |  |
| Biologic treatment |  |  | 8 (17) |  |
| **CCTA Characterization** |  |  |  |  |
| Total burden, mm2 (x100) |  | 0.97 (0.80 - 1.08) | 1.17 (0.96 - 1.58) | **0.005** |
| Non-calcified burden, mm2 (x100) |  | 0.96 (0.75 - 1.08) | 1.16 (0.93 - 1.52) | **0.011** |
| Dense calcified burden, mm2 (x100) |  | 0.01 (0.00 - 0.02) | 0.02 (0.01 - 0.06) | **0.024** |
